# The use of paclitaxel coated balloon (PCB) in acute coronary syndrome of small vessel de novo lesions: an analysis of a prospective ‘real world’ registry

**DOI:** 10.1186/s40064-016-2014-y

**Published:** 2016-03-25

**Authors:** Ahmad Syadi Mahmood Zuhdi, Uwe Zeymer, Matthias Waliszewski, Martin Spiecker, Muhammad Dzafir Ismail, Michael Boxberger, Marcus Ferrari, Imran Zainal Abidin, Wan Azman Wan Ahmad

**Affiliations:** Cardiology Unit, University Malaya Medical Centre, 59100 Kuala Lumpur, Malaysia; Klinikum der Stadt Ludwigshafen am Rhein, Bremserstrasse 79, 67063 Ludwigshafen, Germany; Medical Scientific Affairs, B. Braun Melsungen AG, Melsungen, Germany; Klinik fur Kardiologie, Marien Hospital Marl, Marl, Germany; Klinik fur Innere Medizin I, Universitatsklinikum Jena, Jena, Germany

**Keywords:** Paclitaxel, Balloon angioplasty, Small vessels, Acute coronary syndrome, MACE

## Abstract

**Background:**

Paclitaxel-coated balloon (PCB) angioplasty in small vessel de novo lesions has favourable outcome and appears to be an alternative to stent implantation. However there is limitted data on its use specifically in small vessel acute coronary syndrome (ACS).

**Methods:**

We analyse patients data from the SeQuent Please Small Vessel ‘PCB only’ Registry. It was an international, prospective, multicentre registry which enrolled patients with de novo lesions of small vessel diameter (≥2.0, ≤2.75 mm). Patients were divided into the ACS group and the non-ACS group and comparison made between the two groups. The primary end-point was clinically driven target lesion revascularisation (TLR) at 9 months. Secondary end-points were acute technical success, 30-day and 9-month major adverse cardiac events (death, myocardial infarction or TLR) (MACE) and the occurence of definite lesion and vessel thrombosis.

**Results:**

A total of 447 patients were enrolled for this registry of which 105 (23.5 %) patients were ACS (STEMI and NSTEMI). The procedural success rate was 98.1 % in ACS group. The mean vessel diameter for the ACS and non-ACS group were 2.15 ± 0.36 and 2.14 ± 0.35 respectively. Similar mean lesion length of around 15.5 mm was recorded in both groups. Additional stenting was required in 9.3 % ACS and 6.5 % non-ACS, p = 0.308. Reasons for additional stenting were target lesion related dissection (57.6 %) or non-target lesion stenosis (41.2 %). More than half of the patients had 4 weeks of aspirin/clopidogrel (57.1 % ACS, 60.5 % non-ACS). No significant difference between the ACS and non-ACS groups with regards to the duration and types of DAPT during follow up. At 30-day, MACE rate were (0 % ACS vs 0.3 % non-ACS, p = 0.599). At 9 months TLR rates were (1.2 % ACS vs 4.3 % non-ACS, p = 0.180) and MACE rates (3.6 % ACS vs 5.0 % non-ACS, p = 0.601).

**Conclusion:**

PCB in ACS with small vessel de novo lesions has low 30-day and 9-month TLR/MACE rates comparable to non-ACS small vessels. Thus it appears to be an alternative to stent implantation in the treatment ACS.

## Background

Paclitaxel coated balloon (PCB) angioplasty has proven benefit in the treatment of bare-metal and drug-eluting in-stent restenosis (ISR) (Scheller et al. 2006, 2008, 2012; Harbara et al. 2011; Byrne et al. 2013). The efficacy of PCB in treating small vessel de-novo lesions is also emerging with promising data so far (Ali et al. 2011; Zeymer et al. 2014). However, the data on PCB angioplasty specifically in acute coronary syndrome (ACS) is still lacking. Intravascular plaque rupture and thrombus formation in ACS cast doubt on the effectiveness of paclitaxel drug to be adequately delivered to the vessel wall. However, the increased risk of in-stent thrombosis and restenosis in small vessel PCI with stents (Akiyama et al. 1998; Kasaoka et al. 1998) has made PCB angioplasty, which leaves no intravascular metal a good alternative in theory. Therefore we utilise patients data from a prospective ‘real world’ registry to determine the feasibility of PCB angioplasty in acute coronary syndrome of small vessel de-novo lesions by means of major adverse cardiac event (MACE) and target lesion revascularisation (TLR) at 30-day and 9 months follow up.

## Methods

### Centres

Prospective patients enrolment was done in Germany (20 centres), Malaysia (4 centres), Singapore (3 centres), Italy (2 centres), France (1 centre), Finland (1 centre), Poland (1 centre), China (1 centre) and Iran (1 centre). The study was approved by the individual institutional review boards of the participating centres.

### Materials

In this registry the paclitaxel coated percutaneous transluminal coronary angioplasty (PTCA) catheter based on the Paccocath Technology (SeQuent Please, B. Braun Melsungen AG) was used.

### Inclusion and exclusion criteria

Patients ≥18 years of age with de novo small vessel lesions (2.0–2.75 mm diameter) and stable angina, documented ischemia (non-ACS) and acute coronary syndrome (ACS) were recruited for this registry. There were no patient exclusion criteria except those associated with contraindications for anti-platelet therapy.

### Procedural approach

It was the purpose of this registry to treat de novo lesions with the PCB catheter only without additional stent implantations, according to the German Consensus Group recommendations (Kleber et al. 2011). Predilatation with an uncoated balloon catheter according to the above mentioned recommendation was mandatory (Kleber et al. 2011). In cases of severe dissections or unsatisfactory results post-PCB, the implantation of a BMS was recommended and left to the discretion of the interventionalist.

Vascular access was from the femoral or radial route with recommended diagnostic catheter of at least 5 French in diameter. Due to the ‘all-comers’ nature of this registry, efforts were made to not interfere with established national co-medication recommendations. Dual anti-platelet therapy (DAPT) with acetylsalicylic acid (aspirin) and an ADP receptor inhibitor (clopidogrel, prasugrel, or ticagrelor) was recommended, but at the discretion of the treating physician. An injectable anticoagulant was advised on the basis of local routines in the participating catheterisation laboratories.

### Postprocedural medication

An ADP receptor antagonist (clopidogrel 75 mg/day, prasugrel 10 mg/day or ticagrelor 180 mg/day) was recommended for either 1, 3–6 or 12 months together with aspirin 100–325 mg/day at the discretion of the treating physician.

### Definitions

MACE included TLR, myocardial revascularisation, and death of cardiac or unknown origin. MI was diagnosed with corresponding ECG changes and/or cardiac enzyme elevations according to each institution’s routine diagnostic algorithms.

### End points

Clinically driven TLR (either by re-do PCI or CABG) at 9 months post procedure was the primary end point. Secondary end points were procedural success rate, definite acute/sub-acute vessel thrombosis rates based on the Academic Research Consortium (ARC) criteria (Cutlip et al. 2007) and major adverse cardiac events (MACE) as the composite of TLR, cardiac death, and myocardial infarction.

### Data collection

Baseline and clinical follow-up data were collected through a web based data acquisition system between June 2011 and December 2012. Follow up data were typically collected during routine visits with the treating physician. National principal investigators (one per country) were responsible for the accuracy of their datasets. Source data verification was done when the routinely performed web based plausibility checks indicated discrepancies.

### Statistical analysis

Categorical variables were evaluated with the χ^2^ test. Continuous variables were typically compared with the unpaired two-tailed Student’s t test. In parameters with Gaussian distributions, samples were described using the mean and the SD. SPSS V.20.0 (IBM, Munich, Germany) was used for all analyses at a significance level α of 0.05.

## Results

### Patients demographics and presentation

A total of 471 PCB angioplasties were performed in 447 patients of which 113 (25 %) were ACS (STEMI/NSTEMI). A small proportion of patients (7.2 %) had bail-out BMS stenting. Patients baseline characteristics are shown in Table 1. All clinical characteristics were similar in the ACS and non-ACS groups except for hyperlipidaemia and previous PCI where the non-ACS group had higher rates for both.

**Table 1:** Patient demographics and presentations

Variable	All patients	non ACS	ACS	p value
Number of patients	447	334	113	–
Number of lesions	471	365	106	–
Age (years)	66.1 ± 10.9	66.2 ± 10.2	65.9 ± 12.9	0.761
Male gender	324 (72.5 %)	247 (72.2 %)	77 (73.3 %)	0.824
Diabetes	164 (36.7 %)	128 (37.4 %)	36 (34.3 %)	0.559
Hypertension	360 (80.5 %)	281 (82.2 %)	79 (75.2 %)	0.117
Hyperlipidemia	308 (68.9 %)	244 (71.3 %)	64 (61.0 %)	0.044
History of smoking	169 (37.8 %)	128 (37.4 %)	41 (39.0 %)	0.765
Renal insufficiency	44 (9.8 %)	32 (9.4 %)	12 (11.4 %)	0.533
Prior PCI	238 (53.2 %)	193 (56.4 %)	45 (42.9 %)	0.015
Prior CABG	44 (9.8 %)	34 (9.9 %)	10 (9.5 %)	0.900
Atrial fibrillation	41 (9.2 %)	31 (9.1 %)	10 (9.5 %)	0.887
Age group ≥75 year	113 (25.3 %)	84 (24.6 %)	29 (27.6 %)	0.528
STEMI	41 (9.2 %)	0 (0.0 %)	41 (39.0 %)	–
NSTEMI	64 (14.3 %)	0 (0.0 %)	64 (61.0 %)	–

*PCI* percutaneous coronary intervention, *CABG* coronary artery bypass graft, *STEMI* ST-elevation myocardial infarction, *NSTEMI* non-ST elevation myocardial infarction

### Lesion characteristics and procedural data

The mean vessel diameter for the ACS and non-ACS group were 2.15 ± 0.36 and 2.14 ± 0.35 respectively. Similar mean lesion length of around 15.5 mm was recorded in both groups. The LAD was the most common target vessel for both the ACS and non-ACS group. The distribution of coronary arteries treated were similar for both groups. In terms of lesion characteristics, the ACS group had more acute total occlusion (15.1 vs 7.4 %, p = 0.015) and higher thrombus burden rate (13.2 vs 0.3 %, p = 0.001). The degree of stenosis is also higher in the ACS group (88.5 vs 84.5 %, p = 0.001). Apart from the above, both ACS and non-ACS group have no significant difference in coronary artery complexity. Additional stenting was required in 9.3 % ACS and 6.5 % non-ACS, p = 0.308. Reasons for additional stenting were target lesion related dissection (57.6 %) or non-target lesion stenosis (41.2 %).

### Periprocedure anti-platelet therapy

All patients were on aspirin. Clopidogrel was the most common second anti-platelet drug followed by prasugrel, ticagrelor and ticlopidine. A small minority of patients were on single antiplatelet aspirin (5.7 % ACS and 4.7 % non-ACS). No significant difference in the ACS and non-ACS groups in terms of periprocedural anti-platelet therapy (Tables 2, 3, 4).

**Table 2:** Lesion characteristics and procedural data in ACS and non-ACS patients

Variable	All patients	Non ACS	ACS	p value
Number of lesions	471	353	118	–
Target vessel				
LAD	193 (41.0 %)	154 (42.2 %)	39 (36.8 %)	0.128
LCX	126 (26.8 %)	91 (24.9 %)	35 (33.0 %)	
RCA	94 (20.0 %)	69 (18.9 %)	25 (23.6 %)	
Ramus intermedius	19 (4.0 %)	17 (4.7 %)	2 (1.9 %)	
Other	39 (8.9 %)	34 (9.3 %)	5 (4.7 %)	
Lesion characteristics				
Total occlusion	43 (9.1 %)	27 (7.4 %)	16 (15.1 %)	0.015
Chronic total occlusion	16 (3.4 %)	13 (3.6 %)	3 (2.8 %)	0.714
Thrombus burden	15 (3.2 %)	1 (0.3 %)	14 (13.2 %)	<0.001
Diffuse vessel disease	200 (57.5 %)	156 (42.7 %)	44 (41.5 %)	0.822
Calcification	112 (23.8 %)	83 (22.7 %)	29 (27.4 %)	0.325
Vein graft	9 (1.9 %)	8 (2.2 %)	1 (0.9 %)	0.409
Ostial lesion	48 (10.2 %)	35 (9.6 %)	13 (12.3 %)	0.423
Bifurcation lesion	45 (9.6 %)	35 (9.6 %)	10 (9.4 %)	0.962
Severe tortuosity	45 (9.6 %)	32 (8.8 %)	13 (12.3 %)	0.281
AHA/ACC type B2/C lesion	182 (38.6 %)	133 (36.4 %)	49 (46.2 %)	0.068
Reference diameter (mm)	2.14 ± 0.35	2.14 ± 0.35	2.15 ± 0.36	0.973
Lesion length	15.5 ± 7.0	15.5 ± 7.3	15.6 ± 5.9	0.926
Degree of stenosis (%)	85.3 ± 11.2	84.5 ± 11.4	88.5 ± 10.2	0.001
Procedural data				
Predilation device diameter (mm)	2.02 ± 0.25	2.01 ± 0.24	2.04 ± 0.29	0.303
Predilatation device length (mm)	15.6 ± 4.5	15.7 ± 4.6	15.5 ± 4.3	0.655
Predilatation device pressure (atm)	11.8 ± 3.1	11.8 ± 3.2	11.7 ± 3.1	0.941
DEBs used	478	371	107	–
DEBs per patient	1.07 ± 0.29	1.08 ± 0.32	1.03 ± 0.17	0.114
DEB diameter (mm)	2.33 ± 0.31	2.32 ± 0.30	2.37 ± 0.32	0.138
DEB length (mm)	19.2 ± 4.5	19.2 ± 4.5	18.8 ± 4.3	0.425
DEB inflation pressure (atm)	11.0 ± 3.4	10.9 ± 2.8	11.3 ± 4.9	0.352
DEB inflation time (s)	48.5 ± 15.9	48.5 ± 16.0	48.6 ± 15.6	0.982
Additional stent	34 (7.2 %)	24 (6.5 %)	10 (9.3 %)	0.308
Overall technical success per used device (n = 478)	473 (99.0 %)	368 (99.2 %)	105 (98.1 %)	0.342

*LAD* left anterior descending artery, *LCX* left circumflex artery, *RCA* right coronary artery, *AHA/ACC* American Heart Association/American College of Cardiology, *DEB* drug eluting balloon

**Table 3:** Peri-procedural antiplatelet therapy in ACS and non-ACS patients

Variable	All patients	Non ACS	ACS	p value
Aspirin	447 (100 %)	334 (100 %)	113 (100 %)	–
Clopidogrel	364 (81.4 %)	277 (81.0 %)	87 (82.9 %)	0.792
Prasugrel	41 (9.2 %)	32 (9.4 %)	9 (8.6 %)	
Ticagrelor	8 (1.8 %)	6 (1.8 %)	2 (1.9 %)	
Ticlopidine	4 (0.9 %)	4 (1.2 %)	0 (0.0 %)	
Aspirin + vitamin K antagonists	9 (2.0 %)	8 (2.3 %)	1 (1.0 %)	
Aspirin only	21 (4.7 %)	15 (4.4 %)	6 (5.7 %)	
GP IIb/IIIa inhibitors	10 (2.2 %)	8 (2.3 %)	2 (1.9 %)	0.792

*GP IIb/IIIA* glycoprotein IIb/IIIa

**Table 4:** Duration of dual antiplatelet therapy during follow-up in ACS and non-ACS patients

Variable	All patients	Non ACS	ACS	p value
Number of patients	447	334	113	–
4 weeks aspirin 100 mg + clopidogrel	267 (59.7 %)	207 (60.5 %)	60 (57.1 %)	0.385
3 months aspirin 100 mg + clopidogrel	5 (1.2 %)	5 (1.5 %)	0 (0.0 %)	
6–12 months aspirin 100 mg + clopidogrel	68 (15.2 %)	52 (15.2 %)	16 (15.2 %)	
12 months aspirin 100 mg + clopidogrel	70 (15.7 %)	47 (13.7 %)	23 (21.9 %)	
Aspirin 100 mg + clopidogrel life long	2 (0.4 %)	2 (0.6 %)	0 (0.0 %)	
12 months aspirin 100 mg + prasugrel	3 (0.7 %)	2 (0.6 %)	1 (1.0 %)	
12 months aspirin 100 mg + ticagrelor	3 (0.7 %)	2 (0.6 %)	1 (1.0 %)	
Unknown	29 (6.5 %)	24 (7.0 %)	5 (4.8 %)	

### Duration of dual anti-platelet therapy (DAPT) during follow up

More than half of the patients had 4 weeks of aspirin/clopidogrel (57.1 % ACS, 60.5 % non-ACS). No significant difference between the ACS and non-ACS groups with regards to the duration and types of DAPT during follow up.

### Clinical outcome

Table 5 illustrates the clinical outcome of the patients. The primary end-point of 9 months TLR were 1.2 % ACS versus 4.3 % non-ACS (p = 0.180). Overall, the MACE rate at 9 months was 4.7 % for the entire registry. MACE rates were 0 % at 30-day and 3.6 % at 9 months in the ACS group. The rates were lower as compared to the non-ACS group, but were not significantly different. No cases of target lesion/vessel or non-target vessel thrombosis reported in the ACS group.

**Table 5:** Clinical outcomes in patient populations in ACS and non-ACS patients

Variable	All patients	Non ACS	ACS	p value
Number of patients	447	334	113	–
Patients with clinical follow-up	384 (85.9 %)	301 (90.3 %)	83 (73.5 %)	0.021
Follow-up time (months)	9.4 ± 1.7	9.5 ± 1.7	9.4 ± 1.6	0.875
30-day MACE	1 (0.3 %)	1 (0.3 %)	0 (0.0 %)	0.599
30-day TLR	1 (0.3 %)	1 (0.3 %)	0 (0.0 %)	0.599
30-day MI	1 (0.3 %)	1 (0.3 %)	0 (0.0 %)	0.599
30-day cardiac death	0 (0.0 %)	0 (0.0 %)	0 (0.0 %)	–
9-month MACE	18 (4.7 %)	15 (5.0 %)	3 (3.6 %)	0.601
9-month TLR	14 (3.6 %)	13 (4.3 %)	1 (1.2 %)	0.180
9-month MI	7 (1.8 %)	4 (1.3 %)	3 (3.6 %)	0.168
9-month cardiac death	0 (0.0 %)	0 (0.0 %)	0 (0.0 %)	–
9-month vessel thrombosis	3 (0.8 %)	3 (1.1 %)	0 (0.0 %)	0.356
Target lesion	0 (0.0 %)	0 (0.0 %)	0 (0.0 %)	–
Target vessel	2 (0.5 %)	2 (0.7 %)	0 (0.0 %)	0.457
Non-target vessel	1 (0.3 %)	1 (0.3 %)	0 (0.0 %)	0.599

*MACE* major adverse cardiac events, *TLR* target lesion revascularisation, *MI* myocardial infarction

## Discussion

The definition of small vessel from the literature varies. For our registry, we took the cut-off point of 2.75 mm or less as small vessel. From coronary revascularisation point of view, small vessel PCIs make up a significant portion constituting to about 35–45 % of all PCIs (Zeymer and Scheller 2011). PCI with stenting in small vessels remains challenging as the outcome is poorer compared to PCI of larger vessels. Small vessels have less tolerance to neointimal proliferation post stent implantation (Hausleiter et al. 2002; Agostoni et al. 2005). The risk of ISR in small vessels even with the introduction of DES stents is still considered significant (Meier et al. 2006; Togni et al. 2007). This is in addition to the already known complication of stent thrombosis.

PCB angioplasty in small vessels was first reported in 2010 which showed a TLR rate of 4.9 % in PCB only patients (n = 73) with reference diameter of 2.36 ± 0.18 mm and lesion lengths of 11.3 ± 4.3 mm (Unverdoben et al. 2010). After the initial failure of PICCOLETTO study trial (Cortese et al. 2010) to show a non-inferiority of PCB versus DES, came a larger randomised trial comparing PCB and DES in small vessel de novo lesions (BELLO study) (Latib et al. 2012). BELLO study comprising 182 patients, showed superiority of PCB over TAXUS stents in terms of late lumen loss (LLL) and similar angiographic restenosis, target lesion revascularisation and MACE rates at 6 months. Patients from the BELLO study were then followed up for 2 years and showed better TLR rates (compared to PES TAXUS) at 6 months (4.4 vs 7.6 %), 1 year (6.7 vs 12.1 %) and 2 years (6.8 vs 12.1 %) (Naganuma et al. 2015).

Stent implantation is considered the standard and well established treatment for ACS (Windecker et al. 2014) to improve clinical outcome. However PCI in ACS in small vessels is not spared from the issue of high ISR rate and stent thrombosis. Evidence of PCB specifically in ACS is still sparse. Besic et al. (2015) reported a significantly lower LLL of PCB + BMS combination compared to BMS alone at 6 months but no significant difference in MACE and binary LLL and ISR were noted.

There is now rather convincing data on the efficacy and safety of PCB in small vessels de novo lesions in general (Zeymer et al. 2014). However its use in ACS in particular is still unclear. Ho et al. (2015) reported MACE rate of 4.5 % in PCB primary angioplasty of STEMI at 30-day follow up. Our study shows the outcome of PCB in ACS and non-ACS is comparably similar in small vessel de novo lesions. MACE rate at 30-days and 9-months follow up showed a better trend in ACS than the non-ACS group. MACE at 30 days is 0.3 % non-ACS versus 0 % ACS and 9-months MACE is 5.0 % non-ACS versus 3.6 % ACS. In comparison to other small vessel de novo lesion studies, TLR at 9-months follow up was reported as 3.6 % (PCB only) and 4.0 % (PCB + BMS) (Zeymer et al. 2014) and 1 % (PCB only) and 2.4 % (PCB + BMS) (Wohrle et al. 2012). The mean vessel diameter difference for the two above studies might explain the lower rate of TLR.

## Conclusion

The use of PCB in ACS of small vessel de novo lesion is associated with a low TLR/MACE rate comparable to small vessel PCB angioplasty in general. This suggests PCB angioplasty in ACS is a feasible alternative to stent implantation in ACS.

## Study limitation

Since the purpose of this observational study was the documentation of PCB angioplasty in the clinical routine, event underreporting may have occured. In this context, the procedural details could have been specified in more detail as well. For instance in case of thrombus burden the type of the aspiration device would have been highly desirable. Furthermore, in the case of PCB angioplasty one needs to point out that the learning curve to treat small lesions with PCB only requires positive clinical feedback of each investigator. This entails a learning curve to accept angiographically ‘unpleasing’ results for the benefit of having no implant in a small vessel. However, due to the level of each investigator’s experience a selection bias could not be ruled out in this assessment.
